# Supplementary material for: Intranasal parainfluenza virus-vectored vaccine expressing SARS-CoV-2 spike protein of Delta or Omicron B.1.1.529 induces mucosal and systemic immunity and protects hamsters against homologous and heterologous challenge
Source: bioRxiv. 2024 Sep 13:2024.09.12.612598. Preprint. [Version 1] doi: 10.1101/2024.09.12.612598 (PMC11451599; doi:10.1101/2024.09.12.612598)
Supplement: Supplement 1 [file NIHPP2024.09.12.612598v1-supplement-1.pdf]

bioRxiv preprint doi: https://doi.org/10.1101/2024.09.12.612598; this version posted September 13, 2024. The copyright holder for this preprint (which was not certified by peer review) is the author/funder. This article is a US Government work. It is not subject to copyright under 17 USC 105 and is also made available for use under a CC0 license.

Supplemental figures

Fig S1. Serum antibody responses in immunized hamsters (related to Fig. 2B).

On day 24 or 25 pi, serum was collected from n = 36 hamsters per group. Anti-RBD IgG (left panel) and IgA (right panel) serum antibody titers were evaluated by ELISA using purified RBD antigen preparations specific for the Wuhan-Hu-1 strain. IgG and IgA titers using purified S antigens matching the WA1/2020 or B.1.617.2/Delta or B.1.1.529/Omicron variants are shown in Fig. 2B. Each hamster is represented by a symbol, and medians with interquartile ranges are shown. The limit of detection (dotted line) is 2 $\log_{10}$. One-way ANOVA with Sidak post-test; exact p values are indicated for levels of significance $p<0.05$.

bioRxiv preprint doi: https://doi.org/10.1101/2024.09.12.612598; this version posted September 13, 2024. The copyright holder for this preprint (which was not certified by peer review) is the author/funder. This article is a US Government work. It is not subject to copyright under 17 USC 105 and is also made available for use under a CC0 license.

Fig S2. Anti-S IgG and IgA antibody response in upper airways of immunized and SARS-CoV-2 challenged hamsters (related to Fig. 2A).

On day 21 post-immunization (pi), nasal washes were performed on 18 hamsters per immunized group, picked at random. On day 21/22 post-challenge (pc; equivalent to day 53/54 pi), nasal washes were performed on the six remaining hamsters per subgroup (see Fig. 1B for timeline of experiment). Anti-S IgG (A) and IgA (B) titers of paired nasal wash samples from the same six hamsters per subgroup were determined by ELISA. Note that ELISA titers from the samples collected on day 21 pi are also included in the results from 18 animals shown in Fig. 2A. Each hamster is represented by a symbol. The limit of detection of ELISA titers is 1 $\log_{10}$.

Fig. S3. Serum anti-RBD IgG and IgA responses in immunized hamsters upon SARS-CoV-2 challenge (related to Fig. 5B and C).

On day 23 or 24 pc, the six remaining hamsters per subgroup were euthanized, and sera were collected (see Fig. 1B for timeline of the experiment) for evaluation of the antibody response by ELISA. Serum anti-RBD IgG (A) and IgA (B) titers after challenge (top panels) and fold changes of post-challenge titers over post-immunization titers (bottom panels). N= 6 with the exception of n = 5 for B/HPIV3-immunized/WA1/2020 challenged. Purified preparations of RBD from the Wuhan-Hu-1 strain were used as an antigen in the ELISA assays. Each hamster is represented by a symbol and medians with interquartile ranges are shown. One-way ANOVA with Tukey post-test; exact p values are indicated for levels of significance $p<0.05$.

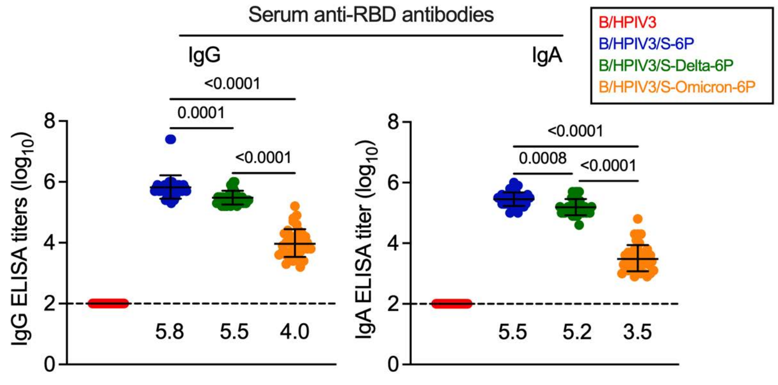

Serum anti-RBD antibodies
IgG
IgA
B/HPIV3
B/HPIV3/S-6P
B/HPIV3/S-Delta-6P
B/HPIV3/S-Omicron-6P
<0.0001
0.0001
<0.0001
IgG ELISA titers (log_{10})
8
6
4
2
0
5.8
5.5
4.0
<0.0001
0.0008
<0.0001
IgA ELISA titer (log_{10})
5.5
5.2
3.5

Figure S1

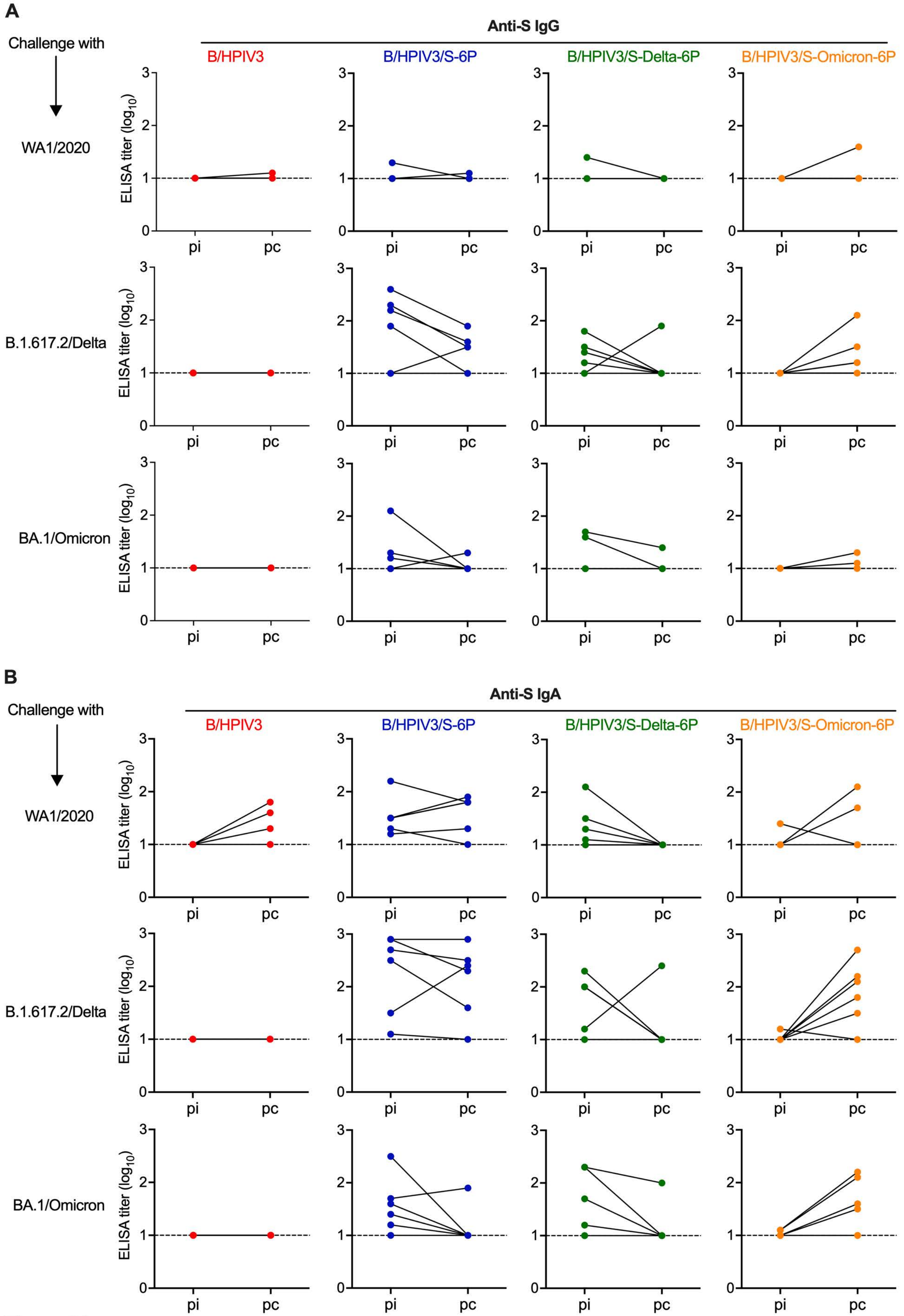
A
Anti-S IgG
Challenge with
WA1/2020
B.1.617.2/Delta
BA.1/Omicron
B/HPIV3
B/HPIV3/S-6P
B/HPIV3/S-Delta-6P
B/HPIV3/S-Omicron-6P
ELISA titer (log10)
pi
pc
B
Anti-S IgA
Challenge with
WA1/2020
B.1.617.2/Delta
BA.1/Omicron
B/HPIV3
B/HPIV3/S-6P
B/HPIV3/S-Delta-6P
B/HPIV3/S-Omicron-6P
ELISA titer (log10)
pi
pc

Figure S2

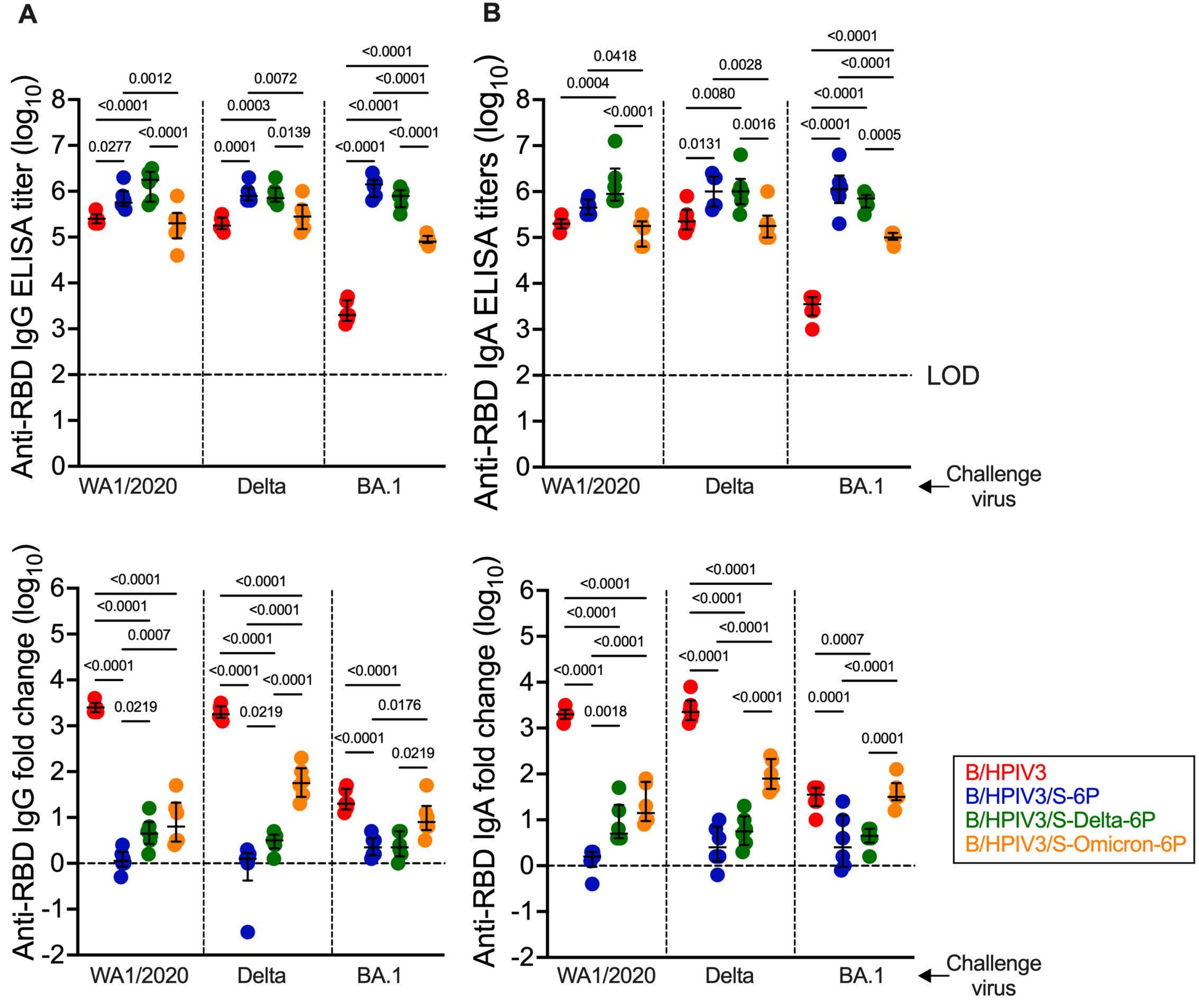
A
B
Anti-RBD IgG ELISA titer (log10)
Anti-RBD IgA ELISA titers (log10)
Anti-RBD IgG fold change (log10)
Anti-RBD IgA fold change (log10)
WA1/2020
Delta
BA.1
LOD
Challenge virus
0.0277
<0.0001
0.0012
<0.0001
0.0001
0.0003
0.0072
0.0139
<0.0001
<0.0001
<0.0001
<0.0001
<0.0001
0.0004
0.0418
<0.0001
0.0131
0.0080
0.0028
0.0016
<0.0001
<0.0001
<0.0001
<0.0001
0.0005
<0.0001
0.0219
0.0007
<0.0001
<0.0001
<0.0001
0.0219
<0.0001
<0.0001
<0.0001
<0.0001
<0.0001
0.0176
0.0219
<0.0001
0.0018
<0.0001
<0.0001
<0.0001
<0.0001
<0.0001
<0.0001
<0.0001
<0.0001
0.0001
0.0007
<0.0001
0.0001
B/HPIV3
B/HPIV3/S-6P
B/HPIV3/S-Delta-6P
B/HPIV3/S-Omicron-6P

Figure S3
